# Health effects of holistic housing renovation in a disadvantaged neighbourhood in the Netherlands: a qualitative exploration among residents and professionals

**DOI:** 10.1186/s12889-024-18500-2

**Published:** 2024-04-16

**Authors:** H.E. Koops-Van Hoffen, Y.M.R. Vendrig-De Punder, F.J. Van Lenthe, F. Borlée, M. Jambroes, C.B.M. Kamphuis

**Affiliations:** 1 Present Address: Department of Human Geography and Spatial Planning, Utrecht University, P.O. 80.115, Utrecht, 3508 TC the Netherlands; 2 Department of Global Public Health & Bioethics, Julius Center, University Medical Center Utrecht, Huispostnummer Str. 6.131, P.O. 85500, Utrecht, 3508 GA the Netherlands; 3 Present Address: Department of Public Health, Erasmus MC University Medical Center Rotterdam, P.O. 2040, Rotterdam, 3000 CA the Netherlands; 4 Department of Public Health, Municipality of Utrecht, P.O. 16200, Utrecht, 3500 CE the Netherlands; 5 Department of Interdisciplinary Social Science, Utrecht University, P.O. 80140, Utrecht, 3584 CH the Netherlands

**Keywords:** Holistic housing renovation, Housing improvements, Socioeconomic interventions, Mental health, Physical health, Renovation stress, Noise nuisance, Resident involvement, Social housing

## Abstract

**Background:**

Holistic housing renovations combine physical housing improvements with social and socioeconomic interventions (e.g. referral to social services, debt counselling, involvement in decision-making, promoting social cohesion). In a deprived neighbourhood in Utrecht, the Netherlands, this paper examined residents’ and professionals’ experiences, ideas, and perceptions regarding holistic housing renovation, its health effects, and underlying mechanisms explaining those effects.

**Methods:**

Semi-structured in-depth interviews were conducted with 21 social housing residents exposed to holistic housing renovation, and 12 professionals involved in either the physical renovation or social interventions implemented. Residents were interviewed in various renovation stages (before, during, after renovation). Transcripts were deductively and inductively coded using qualitative software.

**Results:**

Residents experienced and professionals acknowledged renovation stress caused by nuisance from construction work (noise, dust), having to move stuff around, and temporary moving; lack of information and control; and perceived violation of privacy. Involvement in design choices was appreciated, and mental health improvement was expected on the long term due to improved housing quality and visual amenity benefits. Social contact between residents increased as the renovation became topic for small talk. Few comments were made regarding physical health effects. The interviews revealed a certain amount of distrust in and dissatisfaction with the housing corporation, construction company, and other authorities.

**Conclusions:**

Renovation stress, aggravated by lack of information and poor accessibility of housing corporation and construction company, negatively affects mental health and sense of control. Potential stress relievers are practical help with packing and moving furniture, and increased predictability by good and targeted communication. Social interventions can best be offered after renovation, when residents live in their renovated apartment and the nuisance and stress from the renovation is behind them. Social partners can use the period leading up to the renovation to show their faces, offer practical help to reduce renovation stress, and increase residents’ trust in their organization and authorities in general. This might also contribute to residents’ willingness to accept help with problems in the social domain after renovation.

**Supplementary Information:**

The online version contains supplementary material available at 10.1186/s12889-024-18500-2.

## Background

Poor housing conditions are considered an important contributor of health inequalities between residents of neighbourhoods characterized by low compared to high area-level socioeconomic position (SEP) [1–4]. Improving the physical state of public housing is a promising mechanism to improve the health of lower socioeconomic groups [5–8]. However, public housing residents are often also socially and economically deprived [9–12] and less resourced for handling significant ‘life events’ such as a renovation of their home [13]. Adverse socioeconomic conditions imply recurrent exposure to stressors which may activate mechanisms that affect coping resources and strategies and stimulate appraisal of subsequent stressors as uncontrollable [14]. Holistic housing renovations provide an important opportunity to not only improve public housing residents’ built environment, but also strengthen their capabilities and social environment and support them in multiple life domains (physical, social, economic, health) at once in order to: a) reduce their stress and improve their ability to handle the renovation, which for the contractor also eases the renovation process, and b) achieve added and reinforced health effects [15].

**Fig. 1:**
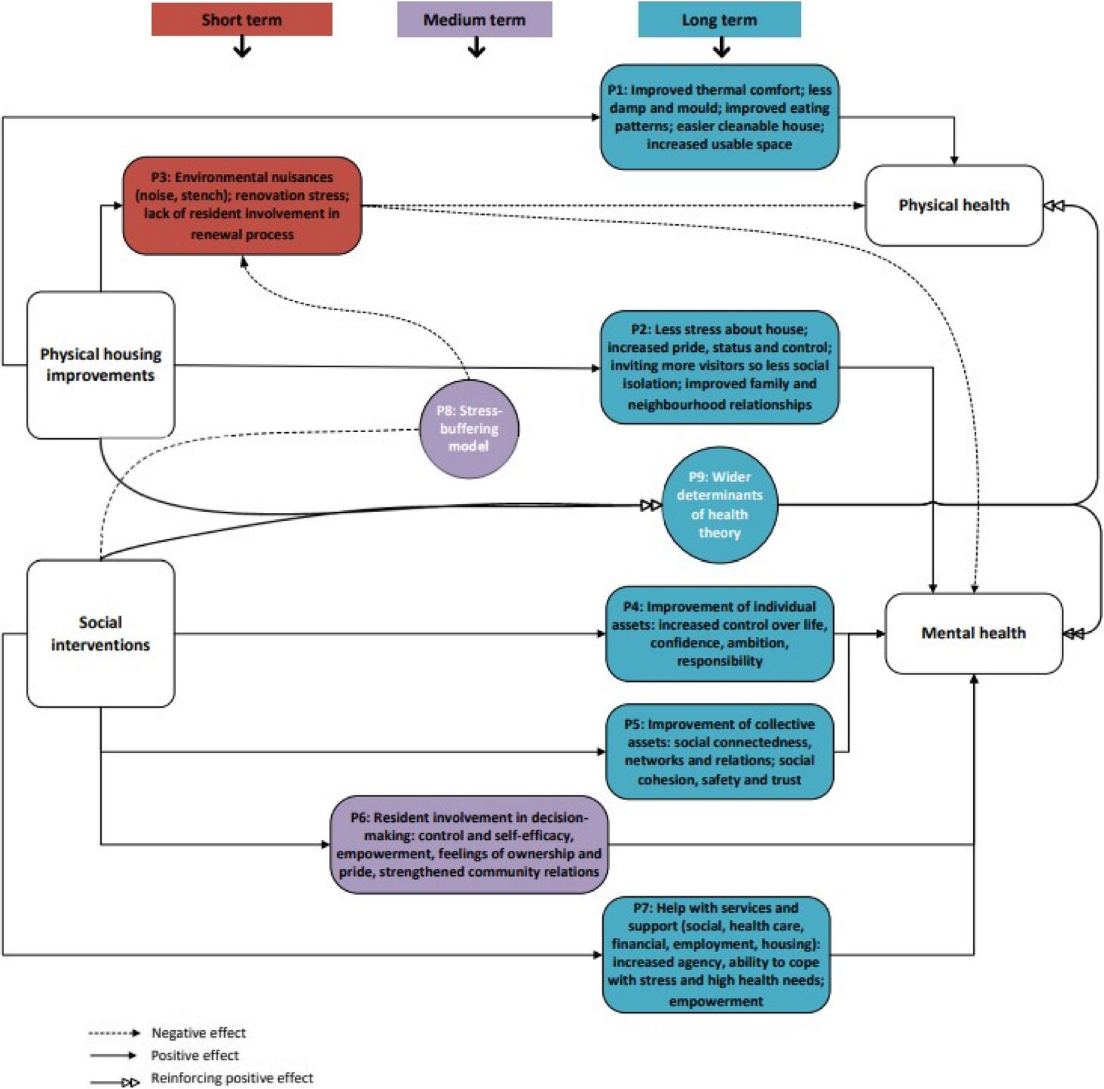
Logic model of the review findings, showing how holistic housing renovation impacts on health (Koops-van Hoffen et al., 2023: p.9)

This article builds upon our recent realist review on so-called holistic housing renovations [15], which provided insight into the underlying mechanisms explaining how a combination of physical housing improvement and social and socioeconomic interventions improving other social determinants of health (e.g. referral to social services, debt counselling, involvement in decision-making, promoting social cohesion) can benefit the health of low-SEP residents and thus contribute to reducing health inequalities. In contrast to ‘regular’ systematic reviews, realist reviews go beyond asking *if* an intervention works, to look for theories as to *why* and *how* a programme works, *who* it works for, and in *what context* or under *what conditions* [16, 17]. We distilled nine pathways, displayed in Fig. 1, via which holistic housing renovations affect health. Physical housing improvements can improve physical (P1) and mental (P2) health (less noise/odour nuisance, improved living environment, less stress, increased pride in the home) and social interventions can improve mental health (P4, P5, P6, P7) (less stress due to increased control, higher empowerment, more social contacts, less financial problems). Further, combining physical renovation and social interventions can have added or reinforced health effects: based on the stress-buffering model, P8 argues that if physical renovation is combined with social interventions, residents will experience less stress and anxiety associated with the renovation, buffering negative health effects described in P3. Based on the wider determinants of health theory, P9 shows that health improvements are greater if improvements are made in multiple life domains simultaneously (additive effect), and that improvements in one domain can strengthen the positive effects in other life domains (reinforcing effect) [15].

Our review pointed to an important gap, showing that existing studies mostly evaluated *either* physical housing improvements [5–8, 18] *or* socioeconomic interventions [19–22] whereas relatively few studies evaluated a *combination* of physical and social renovation. More studies are therefore needed that evaluate holistic housing renovations, to increase insight in the questions how, in which contexts, and for whom holistic housing renovations can improve health.

We aimed to contribute to this gap by evaluating a holistic housing renovation implemented by a social housing corporation in a deprived neighbourhood in Utrecht, the Netherlands. Rather than solely judging *whether* the intervention works, we applied theory-based and realistic evaluation [23], to explain *how, why, and via which underlying mechanisms* holistic housing renovation may contribute to health of social housing residents [15]. In terms of Jolley’s conceptual model for Community Based Health Promotion evaluation [23], our realist review findings [15] filled the ‘evidence bucket’ for our evaluation, containing evidence from academic literature and findings from other research and evaluation [23]. However, we acknowledged the importance of also filling the ‘knowledge bucket’ in our evaluation, by including practitioner and lay wisdom about what has worked before or might be expected to work in this context [23]. Therefore we conducted qualitative, semi-structured, in-depth interviews with residents and professionals involved in the renovation to increase insight into the underlying mechanisms explaining the health effects. To define health, we use the concept of ‘Positive Health’, which is a broader view of health, elaborated in six dimensions which have emerged from research into what people themselves perceive health to be: bodily functioning, mental well-being, meaningfulness, quality of life, participation, and daily functioning [24, 25]. The emphasis is on ‘the resilience of people to adapt to whatever life throws at them and their ability to deal with the physical, emotional and social challenges in life and be in charge of their own affairs’ [24]. This broad conceptualization of health is suitable, as we did not formulate specific hypotheses on how the intervention could affect specific health outcomes, but rather aimed to increase insight into the underlying mechanisms explaining health effects, and potential unexpected or unwanted consequences. Since relatively few studies have looked into possible *short-term negative* health effects of housing improvement and the stressful process residents often go through during physical renovation of their house, we also paid special attention to that. The research question we aim to answer in this paper is: *What are the experiences, ideas, and perceptions of residents and professionals involved in holistic housing renovation in the context of a disadvantaged neighbourhood in the Netherlands regarding its health effects and underlying mechanisms explaining those effects?*

## Methods

### Setting

**Fig. 2:**
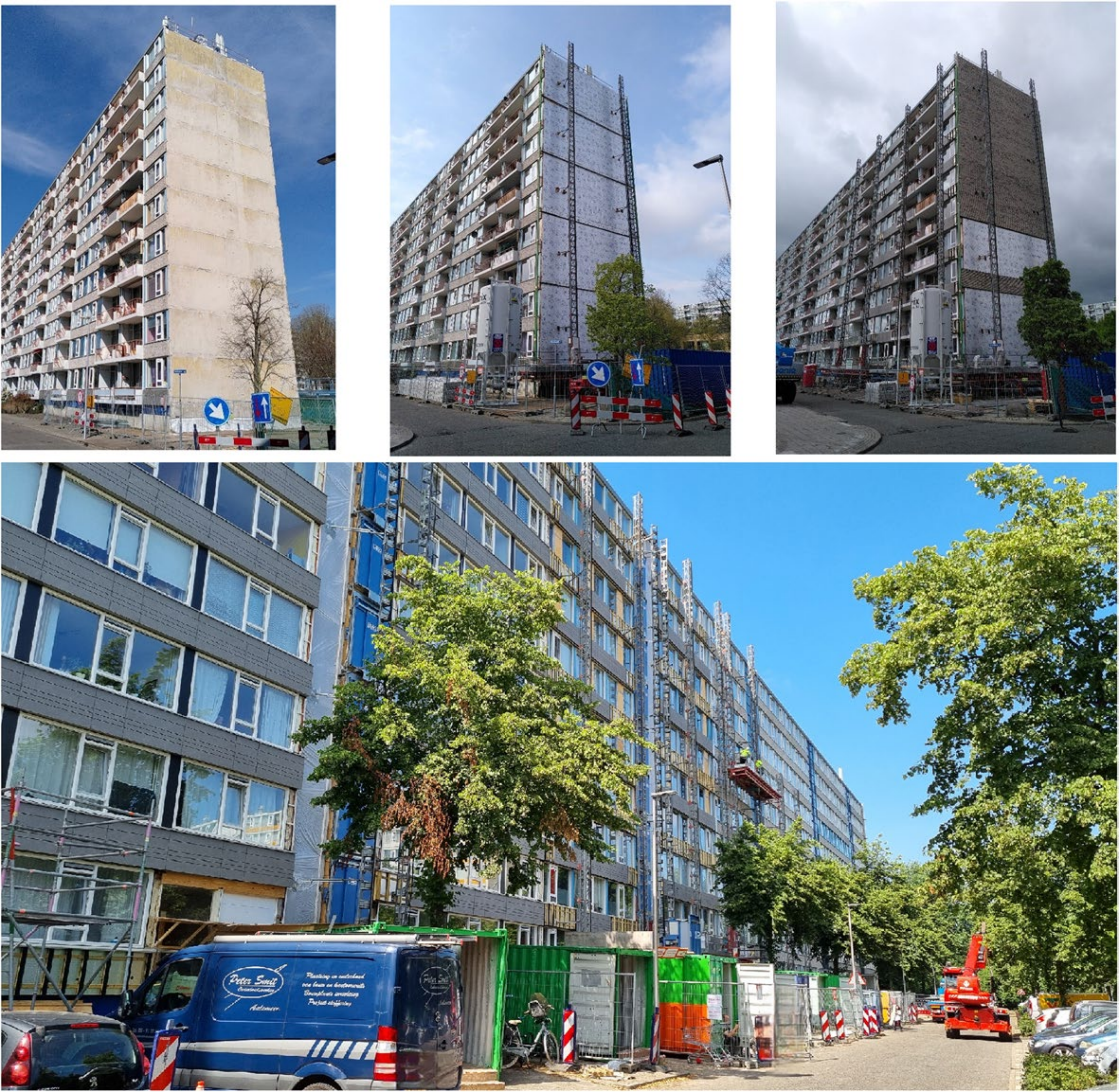
The social housing flat during renovation

This study was performed in a disadvantaged neighbourhood in Utrecht, the Netherlands. The study was part of the IGLO Utrecht study (IGLO is a Dutch acronym for: ‘Towards a healthy urban living environment for all in Utrecht’), in which we cooperated with multiple partners in the neighbourhood to evaluate health effects of various interventions in the living environment [26]. The study population consisted of residents of a social housing flat (174 apartments) undergoing holistic housing renovation, and professionals involved in the implementation of the interventions. Figure 2 shows some photos of the flat during renovation.

Similar renovations are planned for all post-war social housing flats in the neighbourhood over the next ten years. In the Netherlands, social housing is generally only available to low income families; there is a maximum yearly gross income that you can have to qualify, and rents are usually lower than private housing. The Leefbarometer (Liveability meter) of the National Institute for Public Health and the Environment rates quality of life in the neighbourhood under study as insufficient. 42% of its residents rate their health as ‘moderate’ to ‘bad’ and 22% of adults deal with severe loneliness. 42% of the residents have low literacy or are slightly mentally disabled. More than half of the residents in the neighbourhood have a non-Western background. Residents of the neighbourhood have the lowest life expectancy in good perceived health (56,5 years) of the city, which is almost 10 years shorter than the average of an Utrecht citizen (65,0 year) [27].

### Context

The main reason for renovation was that the post-war social housing flat in question was aged and, following national performance agreements for housing corporations, had to be made more sustainable by installing induction cookers to fully get rid of the gas (the building was already linked to the heat network for hot water and central heating before renovation) and improving insulation. At least 70% of the residents had to vote ‘yes’ for the renovation to take place, and 83% did so. Although most residents thus felt renovation was necessary, there were also residents who did not agree, which might have influenced their renovation experience. The holistic housing renovation consisted of both physical housing improvements (summarized in Table 1) and social interventions (summarized in Table 2).

**Table 1:** Physical housing improvements

Houses	Entrance, porches, storage boxes, elevator
• necessary maintenance	• improve building insulation
• replace kitchens older than 15 years	• partial renovation of entrance, new entrance door, mailboxes, intercom and doorbells
• replace bathrooms older than 25 years	• improve safety
• renew toilets and downpipes (where necessary) and sewer connection	• necessary maintenance of porches and stairwells
• replace window frames with new plastic/synthetic frames with insulating glass	• installing new elevator from ground floor to 9th floor to improve accessibility (the old one started on the second floor so people, also with prams or walkers, had to take the stairs first to access the elevator)
• insulating facades (including end facades)	• separate (bicycle)entrance to storage rooms (from the outside)
• apply improved ventilation system	• insulating the ceilings of the storage rooms
• renew / check electricity installation	• insulate the connecting corridor on the 7th floor and adapt it to an open corridor, equipped with external doors to the hall
• adjustments in the field of safety	
• replace balcony fences	
• installation of induction cookers to fully get rid of the gas

**Table 2:** Social interventions planned

Intervention	Organisation/social partner involved
Asset Based Community Development (ABCD): stimulating social cohesion & resident activation	Social welfare organisation
Low-threshold assistance and support with financial debts, financial and social benefits, and steps towards work, activation, or volunteering	Work and Income department, municipality of Utrecht
High-quality general basic care & support with issues on various life domains (financial situation, divorce, domestic violence, residential nuisance) and practical issues	Neighbourhood teams ‘Youth & Family’ and ‘Social’
Occasional help moving/covering furniture, taking curtains down and putting them up again	Construction company
Porch cafés, being present outside the flat with coffee and cookies, offering opportunity to talk about (renovation-related) worries/issues, increasing trust and accessibility	All social partners
Monthly case consultation meetings, anonymously discussing difficult cases and how to help residents effectively	Social partners & Housing corporation
Assistance with move to and practical problems in the temporary apartment (e.g. non-working tv’s, replacing light bulbs)	Home styling organisation
Recruit, train and guide neighbourhood ambassadors (residents with heart for their neighbourhood who want to keep an eye on things, report unsafe situations, and help solve defects in the neighbourhood). 6 ambassadors were recruited, working in rotating pairs	Neighbourhood Approach Foundation

The physical renovation consisted of various stages. Due to different subcontractors and construction flows, apartments underwent various renovation activities over a period of 1,5 years: renovation of gable walls, longitudinal facades, inside of apartments, and porches. This meant residents were not all in the same stage simultaneously. Per strand, residents moved out temporarily for several weeks during renovation of the inside of their apartments. At their return, porch and entrance were not finished yet. The envisioned timeline of the construction work was largely followed, starting in February/March 2021 and ending with the entrances in the 4th quarter of 2022.

Regarding the social interventions, various social partners were involved, focused on different types of help and support (see Table 2) but working together and referring residents to the right partner to help them with their issue. The social interventions and support fall under municipal responsibilities and were thus mostly financed by the municipality. Housing corporations may contribute voluntarily, and possibilities for joint financing are examined at the time of writing. The social interventions were mostly spread out over the period before and during the renovation. After renovation, the Work and Income department of the municipality continued to follow signals and approached residents more actively for six months. Approximately six months after the renovation a party was organised by the social welfare organisation for residents to celebrate the renovated building.

### COVID-19

The interventions were not always delivered as planned due to the COVID-19 pandemic and related government measures, which limited social partners’ possibilities to conduct home visits or organize group activities for residents. Creativity and flexibility was required in their approach, in ways of contacting, reaching, and communicating with residents (e.g. by phone instead of home visits) and in the interventions themselves. Social partners often emphasized this and stated that the impact of the interventions could be expected to be lower than intended. Also, because the study was performed during the pandemic, people spent more time at home due to several (semi)lockdowns and regulations to work from home as much as possible. Therefore, they might have experienced more nuisance from the renovation.

### Study design and analysis

This study aims to explore residents’ and professionals’ experiences and ideas regarding effects of holistic housing renovation on residents’ health, and increase insight into underlying mechanisms explaining these effects. We depart from a realist approach, emphasizing the importance of context and asking not just what works but ‘what works for whom and under what circumstances’ [28, 29]. We therefore chose an explorative qualitative methodology, providing detailed understandings of meanings and lived experiences. Semi-structured in-depth interviews were conducted, combining a deductive qualitative design with an inductive *grounded theory* approach [30]. Our approach was inductive in the sense that during the data analysis *emerging* codes following from the interviews were included in the NVivo analysis. Our approach was deductive, as we build upon the pathways, concepts, and mechanisms as identified in our realist review [15] in two ways. First, we used these to formulate themes and interview questions [see Additional file 1]. For example, residents were asked about their health, perceived home quality (pathway 1 and 2), the renovation process and nuisance and stress residents might experience (pathway 3), and how the social interventions implemented (pathway 4, 5, 6, 7) impacted them. For professionals, after asking more openly which health effects they expected or perceived, we presented a simplified visual display of the mechanisms from our realist review [see Additional file 2], and asked which mechanisms they recognized in residents, whether they could give examples, and what further expectations they had regarding the mechanisms. Second, after the inductive data analysis, we analysed the interviews more deductively by coding the data according to concepts from our realist review, to see whether the data provided evidence (or not) for the pathways and mechanisms from our realist review. Some codes that had emerged from the inductive analysis matched with concepts from our realist review. The interviews took place throughout the entire renovation period (March 2021 – December 2022) in various stages; some residents were interviewed before their apartment was renovated, some during renovation, and some when it was completed, as different mechanisms were expected in every stage. Interviews were held in May – July 2021, March – June 2022, and January 2023.

Transcripts were analysed with NVivo using the constant comparative method [30]. We divided data into fragments, which were compared and grouped into categories labelled with a code (open coding), after which we related categories and subcategories and reassembled data to increase coherence (axial coding). Finally, we sought connections between categories (selective coding). After the first author (the interviewer) coded and analysed the interviews and wrote the draft results, three co-authors (CK, YV, FB) each read 3 to 7 transcripts to ensure no important themes were missing and enhance the quality and reliability of the analysis.

### Recruitment of participants

The interviews were part of an overarching evaluation (IGLO Utrecht study) of the holistic housing renovation. Two members of the research team spent quite some time in the flat for other parts of the study. We used spontaneous conversations as opportunities to invite residents for an interview about their renovation experience. Second, we cooperated with the housing corporation and the contractor’s building foreman. Potential suitable participants received a note in their letter box with the housing corporation’s and university’s logo, explaining they could soon receive a call from a researcher inviting them for an interview. A legally arranged processing agreement enabled the housing corporation to share residents’ phone numbers with the researchers.

Professionals were mostly directly recruited via e-mail invitation. Contact had already been established with most professionals as regular meetings were held with all partners involved in implementing or evaluating the renovation. Some professionals were recruited via the housing corporation.

### Ethical aspects

Residents were informed via an information letter about the study, including the possibility of being invited for a one-hour interview. They were also orally informed by phone by the interviewer inviting them. Professionals received an invite and information letter via e-mail. Participants were requested permission for recording. Transcripts were processed anonymously and unrecognizable. Informed consent was obtained from residents orally, because signed consent can be a barrier for vulnerable target groups. Professionals signed written consent. The study was approved by the Ethics Committee of the Faculty of Social and Behavioural Sciences of Utrecht University.

### Characteristics of participants

We approached 48 residents, established contact with 43, of which 21 were interviewed: 10 before renovation of their own apartment, but while construction work was already going on in other parts of the building, and 11 after renovation of their own apartment (5 while construction work was still going on in other parts of the building; 6 after renovation of the entire building was completed). Reasons for non-participation included lack of time (*n* = 4), lack of interest (*n* = 6), being heavily pregnant (*n* = 2), participating in the fasting month of Ramadan (*n* = 1), or having moved in the meantime (*n* = 2). Others (*n* = 7) kept rescheduling appointments or requesting to call back later but not answering the phone after multiple attempts. We interviewed 12 professionals involved in the holistic housing renovation. Tables 3 and 4 summarize characteristics of participants. Interviews lasted approximately 1 hour.

**Table 3:** Characteristics residents

**Residents**	**Age**	**Sex**	**Interviewed before or after renovation of own apartment**	**Employment status**
R1	47	Male	Before	Unemployed; health insurance benefit
R2	62	Male	Before	Employed (24 h)
R3	63	Female	Before	Unemployed; occupational disability benefit
R4	62	Female	Before	Unemployed; receives debt counselling; under administration / special guardianship
R5	*?*	*?*	Before	*Unknown*
R6	60	Female	Before	Employed
R7	51	Female	Before	Employed?
R8	33	Female	Before	Employed, but currently temporarily receiving health insurance benefit due to pregnancy sickness
R9 & R10	46 & 36	Male & Female	Before	Employed (both parttime)
R11	63	Male	After	Unemployed; health insurance benefit
R12	45	Male	After	Employed
R13	36	Male	After	Unemployed; social assistance benefit
R14	55	Female	After	Employed
R15	39	Female	After	Unemployed; social assistance benefit
R16	47	Male	After	Employed (50 h)
R17	81	Male	After	Retired
R18	*?*	Female	After	Benefit + side-jobs
R19	56	Male	After	Unemployed; social assistance benefit
R20	38	Female	After	Employed (24 h)
R21	45	Female	After	Unemployed; health insurance benefit

**Table 4:** Characteristics professionals

Professionals	Organization
P1, P6, P9	Work and Income department, Municipality
P2, P5, P7	Housing Corporation
P3, P11	Construction Company
P4, P8	Social welfare organization
P10	Center for Housing Research
P12	Neighbourhood team Youth & Family

## Results

Participants talked most about the physical renovation and less about social interventions, which were limited by COVID-19 restrictions. We describe the identified themes one by one.

### Renovation stress: nuisance and damage from construction work, moving stuff around, and temporary moving

The physical renovation was on the foreground of residents’ renovation experience. During renovation, physical and mental health deteriorated due to environmental nuisances and stress. First of all, many residents described noise nuisance starting very early in the morning causing sleep deprivation.

*‘I don’t think it’s bearable. It really is… at six thirty, a quarter to seven, no at six thirty, at half past six, the first truck drives in here. Every day, six days a week, or at least five then, there is a running engine below you… and at seven o’clock the first drill goes into the wall. […] Every day, and then you lie against the ceiling, for as long as… actually already for a year and a half.’ (R9&10)*

Multiple residents described forced lifestyle adjustments: because the noise started early in the morning, they went to bed earlier to get enough sleep. Additionally, at night, loud squeaking noise from the temporary elevators affected sleep quality. The noise nuisance was not just irritating. Residents mentioned headaches, feeling burned out, and becoming so frustrated by the noise and the fact that they could not make agreements about it, that it made them aggressive. Almost all professionals acknowledged the impact of the noise:

*‘The noise is really loud. In a different flat in the neighbourhood, where we also do holistic housing renovation, we have a project apartment, and recently I left that place because I really could not work there anymore. It’s so noisy all the time and it even stressed me out a bit, that I thought: if this is your house, your house which is also your home and your safe haven, and something like this is rumbling past, that’s quite impactful...’ (P4)*

Besides noise, some residents also mentioned irritations from dust, but these were experienced less severe. Another commonly mentioned stress factor was that residents had to constantly move their furniture and stuff (e.g. the content of kitchen cabinets or bookcases) around, or take things apart and put them back together, to make room for the construction company.

*‘You have to stay at home for it, you have to put things aside, put them back, and put them aside again, and put them back again. They take it out one time and a few days later they put it back in again. So you have to put your bed aside and everything, and put it back again, because of course you have to sleep in it again at night. I have to keep living in this house in the meantime.’ (R1)*

Moving to another apartment for several weeks during renovation of toilet, bathroom, and kitchen, was also stressful. On the one hand, residents described how, compared to the period they were living in their own apartment, i.e. during renovation of the window frames, their stay in the temporary apartment was less stressful as it allowed them to escape the noise and dust nuisance. On the other hand, residents experienced stress from having to pack and move their stuff without any help and/or without a car; leaving their own apartment, furniture, and belongings behind while the construction company was at work in their home; or from a lack of comfort in the temporary apartment.

*‘They said: yes, no, it’s just, you only pack a suitcase with clothes and then you move. Well, in practice, it was not how they outlined it, you know. I really had to make sure that he [youngest son] had all of his own stuff, his bath, everything I needed for him. And you know, with older kids, with school, with things, so many things you need to think of, what they need for sports… We really did have a ‘rehousing’ so to speak, for 5 people.’ (R20)*

Some residents would rather have stayed in the temporary apartment for months instead of weeks, as they also experienced nuisance from construction work in adjacent apartments and from renovation of window frames. Moreover, they had often heard stories from acquaintances, friends, or extended family about other housing corporations that temporarily moved residents for a few months and did all the work at once, so it was finished when residents returned. Being exposed to construction work for almost 1,5 years, residents’ stress was aggravated by the long duration of the renovation and long period of exposure to environmental nuisances. This was also acknowledged by professionals:

*‘Dust, noise, construction workers in and out, taped walkways that have to be left in place for a long time, things like that. Also the entrance, a messy entrance, or let’s say primarily consequences of construction activities. That causes nuisance. And the duration. I think anyone could manage about a month, but this is a year and a half, in the immediate environment.’ (P7)*

Additionally, residents described how, when returning to their renovated apartment, their home was filthy and dusty, not cleaned properly, and there was damage to floors and walls due to construction work.

*‘They had damaged all my walls. Literally destroyed all my walls! […] You have already been through so much with that whole renovation […] Finally, I may enter my own home again, and then you end up in a ruin!’ (R19)*

*‘I suppose all that dust in the house is also not really healthy either. And especially when I returned here, when the renovation was over, it was really a terrible mess in the house.’ (R18)*

Some complained they were not reimbursed properly for the damage, nuisance and long stressful process, and that the renovation forced them to make additional costs they were not always able to make. For example, one resident explained her rheumatism required a different, non-standard faucet, which she had to pay for herself with her limited budget.

### Renovation stress: lack of information and communication and the mechanism of control

Residents’ stress and anxiety during renovation was also associated with not knowing what was going on and lack of personal control/influence. Residents described that their stress was often caused or worsened by a lack of information and communication about the renovation and unclarity about what was going to happen when and what was expected from them. For various reasons, the approach sometimes deviated from how it was planned and communicated in advance.

*R1: ‘At the moment, my stress level is a bit higher due to the renovation.’ Interviewer: ‘Can you explain that? Where does that come from?’ R1: ‘Lack of information, lack of clarity. Change of plans. We would first, at first it was the plan to just leave your home for six weeks and in those six weeks everything would be done. So they changed that. […] Now they are going to do the window frames and insulation at the front. Then at the back. And then they come back one more time to do the interior. […] When they do the inside stuff, I’ll be out for a few weeks.’*

One resident mentioned lack of information regarding different apartment types within the building: there was only detailed information about how the bigger apartments would be renovated, not about the smaller ones. What also caused stress were changing schedules which required instant rescheduling of appointments for residents, as they needed to be at home to let workers in their house. Residents experienced little acknowledgement that this caused discomfort for them as they needed to reschedule their work hours or doctors’ appointments on short notice. Residents also described that when they had complaints, e.g. about nuisance or broken agreements, it was not always clear who was responsible and the housing corporation and construction company were poorly accessible and often pointed at each other or referred residents to each other, sending them from pillar to post, further limiting their perceived control. Whereas most residents talked about a lack of information and communication causing stress, some residents explained how information they received slightly reduced their stress.

*‘So yes, once that information was there, I slowly started to feel more and more reassured […] From the housing corporation and construction company, those newsletters we are receiving now, I think that is very good. Those are those kind of leaflets. I then keep them well and they also contain the schedule and what is about to happen.’ (R4)*

Professionals recognized the lack of control residents dealt with and reflected on the importance of informing and involving residents in the renovation to reduce stress:

*‘That control over your own life, I very much notice that people have the feeling that they have lost control for a while. People just don’t know what they can expect regarding the renovation, schedules that keep changing…’ (P4)*

*‘When I don’t explain it to someone, and I just let it happen, then I know for certain that that person is going to get a lot of stress. And of course, we often deal with people who already have a lot of problems, so yes, you certainly need to involve them.’ (P1)*

### Renovation stress: violation of privacy and not feeling at home anymore

Residents’ feelings of control were also affected by a perceived privacy violation. Some residents did not feel at home in their own home anymore. One resident described how construction workers burst into his house without making an appointment first, and how he felt being watched constantly by workers moving up and down along the windows on construction elevators. Others found it difficult to leave their house and belongings behind during the temporary move, fearing theft or damage. One resident experienced a lack of respect for her home and belongings as workers did not lock the door behind them when she could not be present once. Some professionals also reflected on the privacy violation experienced by residents:

*‘You can’t find peace in your own home. It’s just really an invasion of your privacy. That is also what people say: all those people, all those construction workers around me […] that walking in and out, but also how they handle the stuff, you know: hitting the wall hard with packages of window frames, a dent in the wall, yes we will fix that later... Whereas it’s about your home, it’s about your own place, you know […] I am always very careful, almost autistic with my stuff, so I recognize it and I can relate to it quite well. I myself also don’t like people stamping loudly through the room, with those work shoes. I was there once, then someone comes in, stamp, stamp, stamp… And look, he can’t help it, because he’s just working hard, I get that too, but well…’ (P9)*

### Double burden and fatalism

Some residents experienced a ‘double burden’ because the renovation came on top of their already difficult living circumstances. For example, a father described how the renovation had been an enormous burden: all the uncertainties (e.g. about asbestos), the dust being a problem for his children with asthma, the temporary move that brought a lot of stress, and that all together with his wife being sick and him doing physically hard work for 50 h a week. A mother described how the renovation was tough on top of their already challenging personal situation being in the middle of a diagnosis process of a child with a disability. Some residents mentioned the renovation forced them to make additional costs, e.g. to hire people for help or fix damage to floors or walls, which caused stress due to lack of money. Residents seemed somewhat fatalistic about the situation: whereas the renovation was very heavy, they were not always very angry about it: the renovation just happened to them, which was very annoying, but they couldn’t change it anyway. And most were happy with the result.

### Resident involvement

Residents appreciated being involved in design choices regarding the type or colour of the new tiles, counter tops, and kitchen cabinets:

*‘I will get a new bathroom, a new toilet, a beautiful new kitchen. Especially the counter top, you can choose, you can take multiple cabinets. And I thought: yes, that’s what I want! […] Tiles too, I have already chosen.’ (R3)*

However, some residents felt they were not always really listened to:

*‘What I find disappointing: I had specifically indicated to [housing corporation] how high I wanted the kitchen cabinets to be. Do you want 90, 92, 94 [cm]? I said: 90. Told them twice. Received confirmation twice. And then they install it at 92. So all of my things I had made, like this, an extra shelf here below… I saw: that’s not right. I measured it when I just returned here. I said: here it is, in black and white: 90. And this is 92. I said: well, never mind, I’ll come up with something, otherwise the entire kitchen needs to come out again, and I wanted to move back into my house…’ (R19)*

Another resident described how the garden his wife had created with various plants was removed without involving him. Residents indicated they would have appreciated being involved more regarding choices that could contribute to their living comfort, e.g. wanting to use unused space in the toilet to increase usable space in the bathroom and place a bathtub; not being allowed to drill holes in the new window frames after renovation while sun screens and shutters were removed, which was experienced as a deterioration also affecting health since it became harder to keep the heat out. Another resident mentioned not being allowed to drill in the new bathroom tiles, preventing her to install a glass shower cubicle.

Professionals also reflected on the importance of involving residents, e.g. regarding design choices, for them to feel seen and heard, especially for residents in deprived areas who are relatively poor or low on the societal ladder.

*‘People really like to be heard. People really enjoy being able to have their own say. They already feel so low on the ladder in so many ways. They never feel taken seriously. I think that’s really important, that you really listen to people like: what do you want?’ (P10)*

### Improved housing quality and visual amenity benefits

Whereas during renovation mental health deteriorated, we found indications of (expected) health improvements after renovation. Before renovation started, residents looked forward to living in their renovated apartments and expected visual amenity benefits from their own and other renovations in the area, which could contribute to mental health and wellbeing.

*‘I didn’t want to stay anymore. I had already given up hope until I heard: they are going to start now. And when I was allowed to choose a kitchen, toilet, because my toilet isn’t tiled, it’s still the same old toilet, bathroom, everything… So I had already given up hope a little bit, like I was not going to last any longer. But when I heard this, then… I became hopeful again.’ (R3)*

After renovation most residents were happy with their new kitchen, induction cooker, and bathroom, and some were prouder of their home and building:

*‘I also really notice when visitors come, you know, when family comes over, that they also say: wow, so, well, this is different from how it was before. That you also hear it from others: yes, now it looks good, now it is at least neat and nice and fresh, you know? So yes, I think now, when you enter, you have a different feeling than how it was in the old state.’ (R20)*

Professionals similarly described how renovation can improve mental health:

*‘If you are in a nice environment where it is neater, where it is tidy, you will also feel more comfortable. If you’re not in a depressed room with mould on the walls [...] Fresh walls, fresh air around you, beautiful tiles on the wall, you know, yes, that does something to people […] There are people who say: it’s so incredibly nice. And only now they realize in what kind of mess they actually lived before, and what that does to you.’ (P10)*

They described a clear trend that during renovation residents’ health first deteriorates, as they go through a rough patch as the basis of their life, a safe and secure home, is invaded in and tampered with, whereas after renovation their health improves, they are proud of how clean and tidy their house is, and the appearance of both the outside and inside of the building makes them feel better.

Relatively few explicit comments were made regarding physical health effects of the renovation. Some residents expected insulation and improved indoor climate would improve health, primarily for people with poor health, respiratory problems or joint problems. Others were happy with the insulation due to reduced noise nuisance from outside or less draughts and improved thermal comfort, which can affect both mental and physical health:

*‘Look, this [pointing to new window frame], is actually pretty sound proof, whereas the previous one wasn’t. So you hear the beeping of those reversing trucks a bit less, so that’s an improvement in quality, you hear children screaming less… And my big enemy, the sweeper truck of the municipality that arrives at half past six in the morning and thunders through the street, you don’t hear that that much either.’ (R9)*

*‘Renovation is good, because it is cold here in the winter, we suffer from draughts. At my sister’s, she also lives in this neighbourhood, also in a flat from the same housing corporation, it is already renovated, and there you notice that too. There the draughts are less, it is warmer.’ (R8)*

Some residents commented on improved mechanical ventilation in the bathroom and kitchen, decreasing moisture and mould. Others complained about increased draughts due to the new ventilation vents, or window frames that were not properly sealed. Some professionals expressed concerns about residents’ understandings of the workings of upgrades, e.g. the new ventilation system, and how this can affect their health.

*‘Sometimes I wonder […] To what extent do they handle the window frames properly, to what extent do they ventilate the home properly? Will they even ventilate? Or are they going to close all those ventilation vents, which I often see. That also has to do with health. […] They receive a booklet that explains everything, how it works, but I have the feeling, not everyone, but some think: it will all work out […] That is one part that concerns me a bit regarding residents’ health. I think a few people think: what kind of booklet is this? And they throw it in the waste bin. […] They get a completely new system, but they also have to use it in the right way.’ (P11)*

Residents were asked whether their house was easier to clean after renovation. Some did not experience any changes or only described how during renovation their house was harder to clean due to increased dust levels. Others described how new materials, e.g. new tiles or induction cooker, were easier to clean. One resident specifically mentioned how this influenced her and her family’s health and wellbeing:

*Interviewer: ‘What do you find the nicest change?’ R20: ‘Oh, a thousand percent the shower. I mean, that’s where you want to get it really clean, you know, I bathe my kids there. […] Now of course everything in it is new, so yes, that’s just nicer and easier to work with when cleaning: new tiles, new sanitary facilities. […] My husband is very sensitive to dust mites, allergies. He also notices a clear difference, now that the house is simply better insulated and easier to keep clean, as we said. Yes, then he really notices that the quality of the air is improved, and the extent to which he suffers from that allergy is clearly less now.’*

### Increased social contact

The physical renovation and the fact that they were all in the same boat together increased residents’ social contact with neighbours:

*‘Because you already have more to say to each other like: oh, do you know how that goes? And: oh, that gable wall is going to look pretty nice, isn’t it? Oh, how do they actually do that with those windows, that’s strange to see! Yes, very simple conversations, but because you all see that renovation happening now and you know that you will also get that in your own apartment or flat, that provides an extra connection with each other. Because of that renovation you have a common bond, you may encounter the same kind of problems, or you have the same questions or fears, like I had.’ (R4)*

Some residents mentioned neighbours helping each other, e.g. with putting up wallpaper.

### Timing of social interventions

The initial idea behind the holistic housing renovation was that, before the physical renovation started, social partners could help residents with their problems in other life domains, so they would have less problems simultaneously and handle the renovation better. However, the interviews showed the importance of timing the social interventions right. Before renovation, residents experienced stress about the upcoming renovation, which triggered more ‘practical’ help from social partners, e.g. in providing information and clarity about the renovation and what was going to happen, reading news letters about the renovation together with residents, explaining things to them, and helping residents with practical issues they worried about, like how to handle the move to the temporary apartment. Professionals anticipated on this and instead focused on just being there for residents, showing their faces, offering help, and reducing residents’ stress. This facework seemed to increase residents’ trust in social partners involved, which might on the long term increase their willingness to accept help with socioeconomic problems they might have.

*‘For example, we also have people who found it difficult, who could not estimate, for example: yes, but when I have my renovated house, what will it look like? Then someone from the neighbourhood team at some point just made a drawing on the floor on paper like: this is your surface, where are you going to put your things? Just very concrete. That kind of things are of course nice, because then you gain confidence and trust and next time that person will think: hé, that Piet from the neighbourhood team, I’m going to call him again next time. And then you have their trust and you hope that the rest will follow.’ (P1)*

Additionally, increased contacts between social partners and residents during renovation might also be a good starting point for community building later on:

*‘In the short term I really think that we have been able to remove a bit of stress people had, and the part of really community building and stuff, that is really… the social renovation has actually been a great way for us to be among the people and to come into the picture. And then we can gradually intertwine and connect the contacts we have made during the renovation.’ (P4)*

### Distrust in and dissatisfaction with the housing corporation and other authorities

The interviews revealed a great level of dissatisfaction and distrust of residents in the housing corporation, municipality, and other authorities, caused by a number of factors. Pre-renovation residents commented on poor quality of their house and deferred maintenance and lack of involvement of the housing corporation in this respect, which was often related to the upcoming renovation.

*‘I had already said a few times: can’t you do something about my bathroom? Because the drain is not working properly, it did not open so I couldn’t clean it properly. And then the housing corporation told me: sir, you have to live with it for another year or two. If you just pour a lot of drain unblocker or something through it, two or three times a year, then it will drain.’ (R19)*

Besides deferred maintenance, other factors contributing to feelings of dissatisfaction and distrust were: changing schedules causing feelings of insecurity and lack of control, the renovation approach taken, and the nuisance and damage experienced. The poor accessibility and problem-solving behaviour of the housing corporation and construction company, and the fact that they kept sending residents from pillar to post or blamed one another for problems residents experienced, further aggravated feelings of dissatisfaction and distrust.

## Discussion

This paper examined residents’ and professionals’ experiences and perceptions regarding holistic housing renovation, its health effects, and underlying mechanisms explaining those effects. Residents' renovation experiences were primarily negative due to the great amount of renovation stress caused by nuisance from construction work (noise, dust), having to move stuff around, and temporary moving; lack of information and control; and perceived violation of privacy. These stressors and challenges were acknowledged by professionals. Residents were positive about their involvement in design choices. Both residents and professionals expressed expectations of mental health improvement on the longer term due to improved housing quality and visual amenity benefits. The interviews also showed increased social contact between residents as the renovation became easy topic for small talk. Also, interview participants shared ideas about the right timing of social interventions: they can best be offered after renovation, whereas the renovation itself can be used by social partners to show their face, offer practical help, and build residents’ trust. Relatively few explicit comments were made regarding physical health effects of the renovation. Last, the interviews revealed feelings of distrust in and dissatisfaction with the housing corporation, construction company, and other authorities.

The findings from this interview study confirm pathways as identified in our earlier realist review [15] and are in line with results from similar qualitative studies. During renovation health deteriorated due to environmental nuisances and renovation stress caused by i.a. temporary moving. This confirms pathway 3 from our realist review [15] and findings from studies that looked into (health) effects of physical housing renovations among elderly and people with a disability [31, 32]. Our study showed that these mechanisms might be more universal, or related to time spent at home (as our participants spent a lot of time at home due to the COVID pandemic), rather than to age or disability per se. Furthermore, stress and anxiety during renovation came forth of not knowing what was going on and a lack of personal control/influence, whereas involving residents in decision-making (e.g. regarding the choice of new kitchen tiles) increased feelings of control, self-efficacy, empowerment, ownership and pride, improving mental health, confirming pathways 3 and 6 [15] and findings from Bal et al. [33] and Allen [34]. Similar to our study, Granath et al. [35] found respondents had negative emotions connected to the renovation and relocation process, e.g. worries about the (lack of) information before the renovation started, or not wanting to live through a renovation in their present apartment, and that “the renovation project in itself, with the disturbances and stresses it brings, are a large cause for people to move” ([35], p.5). Our findings further indicated that after renovation, mental health might improve due to improved housing quality and visual amenity benefits, which decrease stress about housing problems and increase feelings of pride, confirming pathway 2 [15] and findings from Orlando-Romero et al. [36] highlighting the psychosocial component of people’s relationship with their house.

Whereas social interventions like Asset Based Community Development show potential to increase social contact between neighbours (pathways 4 and 5) [15], our interviews showed social contact between neighbours primarily increased due to the *physical renovation* rather than through social interventions implemented. This might be explained by COVID-19 restrictions limiting implementation of social interventions intended to increase contact between neighbours, e.g. neighbourhood activities. Social interventions implemented were thus more remote and primarily implemented on an individual base, e.g. checking whether residents had issues they needed help with or would like someone to check whether they were using all financial arrangements they were entitled to. Initially, and in line with the stress buffering model (pathway 8) [15], the idea was that before the renovation started, the social partners could help residents with their problems in other life domains, so they would have less problems simultaneously and handle the renovation better. However, the interviews showed the importance of primarily practical help in preparation of and during the renovation, while people experience more room for help with other issues *after* renovation. Furthermore, our findings confirmed the importance of resident education about changes and upgrades to their home, e.g. the new ventilation system, and how to use these in such a way that it contributes to their health [33, 37–42]. Careful consideration is required regarding how this information is provided, e.g. complementing written brochures with oral explanations and demonstrations [33, 42].

### Limitations

We found little evidence regarding pathways 4, 5, 7 and 9 from our realist review, which are focused primarily on social interventions and/or longer term effects [15]. The COVID-19 pandemic limited social partners’ possibilities to implement social interventions as planned, and changed the character and timing of the social interventions, which might also explain why many residents were unaware of the social interventions. Pathway 3 on the other hand came out extra strong, as the pandemic forced residents to stay at home, limiting their possibilities to flee from construction work nuisances.

We found little evidence regarding pathway 1 from our realist review [15] which focuses on physical health effects of housing improvements. This might be explained by the timing interviews were conducted. Residents were mostly interviewed either before or shortly after their own apartment was finished, but while other parts of the building were still under renovation. A few residents were interviewed shortly after the entire building was completed, but physical health effects might be more likely to occur on the long term. We were limited by research funding being only available for a few years, so a long term measurement would have to take place outside of our study period. Studies have shown potential for increasing residents’ wellbeing as part of energy renovation, in terms of satisfaction (e.g. improving perceived thermal comfort, air quality, sound/acoustics, accessibility and contributing to a more positive perception of the buildings’ expression), but thereby also improved perceived health [43, 44]. More research is therefore needed into long term health effects of holistic housing renovation, as both effects of physical housing improvements but also effects of social interventions might be more visible in the long term.

Methodologically, interviews might be regarded as inadequate to assess or document changing objective health due to the interventions as they are limited in their ability to measure ‘hard’ effects. However, we aimed to document *experienced* and *perceived* (changes in) health and well-being rather than *actual* changes in health. Moreover, with interviews, we could gain better insight into underlying mechanisms and what works for whom, when, and under what circumstances, and increase insight into effects on unexpected health outcomes (for which no items would have been included in a survey) and potential undesirable effects. Since this was an exploratory study, the advantages of interviews outweighed the disadvantages.

The number of interviews can be regarded a limitation. One can argue whether 33 interviews are enough to make generalisations. Although saturation was reached in this particular study, we recommend that more similar studies are conducted, also in different local contexts, to strengthen the evidence base and the generalizability of our results and conclusions.

### Recommendations for practice

Our findings have several implications for holistic housing renovations in practice. According to the stress buffering model, combining physical housing improvements with socioeconomic interventions can work as a buffer against negative health effects of physical renovation, since social help and support can relieve residents’ stress, diminish overall levels of disadvantage, and help them overcome the renovation better [15]. Our findings however showed that participation in social interventions may just be too much for residents in the period prior to the renovation, as then residents are experiencing a lot of stress and worries about the upcoming renovation and lack peace of mind to deal with other problems they face. We argue that prior to the renovation, residents benefit more from practical help and support and clear communication about the renovation. Residents like to be involved in the process, e.g. have a say in the type of tiles they get in their new kitchens. Practical support (e.g. with cleaning out their house, disposal of bulky waste like old furniture), clear communication and some level of involvement in decisions will increase residents’ perceived control and reduce stress and worries about the (upcoming) renovation. As Allen also concluded: “*the opportunity to exercise an appropriate level of control had a clear relationship to health outcome, in some cases, by reducing stress*” [34]. Moreover, this approach might increase residents’ trust and confidence in the organizations, professionals, and authorities offering this practical help, which on the long term can increase their willingness to receive help with other, socioeconomic problems. Social partners and housing corporation are recommended to first invest in their relationships with residents and to postpone offering socioeconomic help and support until after the renovation. After the renovation, residents live in a clean, tidy, renovated apartment and have less stress about housing problems or the upcoming renovation. This might be a better starting point as residents have more room in their heads and life to accept help and support on other life domains. Additionally, social interventions should ideally continue after the physical renovation, since community building and strengthening social cohesion have a long-term horizon.

Our findings also showed that, in order to reduce renovation stress, it is important to closely examine what a suitable period is for a temporary stay elsewhere, and that this should depend on the total amount of nuisance that can be expected at a certain time point in the renovation process, not only due to work on the inside of apartments, but also due to construction work taking place outside of residents’ own apartment, e.g. in the surrounding porches. This requires tailoring these decisions to specific relatively more vulnerable residents, e.g. pregnant women, families with children, or retired elderly who spend large parts of their days indoors. As Allen also concluded, people experience housing renewal differently, the issue of personal control is more important to some than to others, and some residents suffer adverse health effects while others do not [34].

Tailoring is also needed regarding financial compensations to ease the pain both from actual damage to apartments as from everything else the renovation entails in terms of ‘suffering’. In their opinion, residents were not sufficiently compensated financially. It is important for housing corporations to realize that for residents, their house does not feel like home for 1,5 years and they often have to make additional costs. It is worth considering a form of compensation that can diminish negative health effects of renovation.

Furthermore, our findings showed the importance of clear and targeted information and communication (e.g. regarding schedules, expected noise nuisance, and the workings of new upgrades and how to use these) and some level of resident involvement in decisions (e.g. regarding colour or type of tiles), which can contribute to perceived control.

## Conclusions

Physical renovation causes a great deal of stress among residents in deprived neighbourhoods, who are often already in a vulnerable situation. The stress is caused by noise and dust nuisance from construction work, temporary moves, and the unpredictability of the renovation, which for residents is partly aggravated by a perceived lack of information and communication. This has negative effects on mental health and sense of control over one’s life. There are factors that can contribute to reducing this stress, such as practical help with packing things and moving furniture, but also increasing the predictability of the renovation by good and targeted communication. The momentum for social interventions, as part of holistic housing renovation, is not before the renovation starts, when many residents experience stress about the upcoming renovation, but rather after renovation, when residents live in their renovated apartment and the nuisance and stress from the renovation is behind them. Social partners can use the period prior to the renovation to show their faces, offer practical help to reduce renovation stress, and increase residents’ trust in their organization and authorities in general. This might also contribute to residents’ willingness to accept help with problems in the social domain after renovation. This study is one of the first to qualitatively evaluate holistic housing renovation. More similar studies are needed in different contexts to increase insight in the questions how, in which contexts, and for whom holistic housing renovations can improve health.

### Supplementary Information

** Additional file 1.**  Interview guide. Interview guide with questions used for the in-depth interviews.

** Additional file 2.**  Simplified visual display of the mechanisms from our realist review. Simplified visual display of the mechanisms from our realist review Description of data: Simplified visual display of the mechanisms from our realist review, used in the interviews with professionals.

## Data Availability

The datasets generated and/or analysed during the current study are not publicly available due to individual privacy protection but are available from the corresponding author on reasonable request and under restrictions.
