# Supplementary material for: Health effects of holistic housing renovation in a disadvantaged neighbourhood in the Netherlands: a qualitative exploration among residents and professionals
Source: BMC Public Health. 2024 Apr 16;24:1056. doi: 10.1186/s12889-024-18500-2 (PMC11020185; doi:10.1186/s12889-024-18500-2)
Supplement: Supplementary file 1 — Additional file 1. Interview guide. Interview guide with questions used for the in-depth interviews. [file 12889_2024_18500_MOESM1_ESM.pdf]

Interview guide

<u>Interview guide for interviews with residents ***before*** renovation of their own apartment</u>

Introduction and demographics

Can you introduce yourself? Can you tell me something about yourself?

- How long have you been living here?
- Who do you live here with?
- Do you have a paid job and what kind of job do you do?
- How old are you?

Health – meaning and definition

The research we are doing is about how various changes in the neighbourhood, such as the renovation of your flat, influence the health of the people living here. Before we actually talk about the renovation, I am curious:

What do you understand by health? When I say the word 'health' what comes to your mind? It can be anything, you can just say what comes to mind.

When would you define someone as 'healthy'?

When would you define someone as 'unhealthy'?

How is your own health? Can you tell me something about that?

What do you do for your health, to stay healthy?

Renovation

Now let's talk about the renovation, because that is of course what you are dealing with now.

What do you think about old flats in the neighbourhood being renovated? What is your opinion about that?

What do you think of the renovation of your own flat?

What do you think of your house?

How would you describe the quality of your home? What do you like or dislike about your home?

Are there certain problems in your home that bother you or that affect your health and wellbeing or the way you feel?

How stressful is your living situation right now? What things stress you out the most?

How does the renovation affect your health and how you feel?

Do you think that is different during the renovation than after the renovation when the houses are finished?

For the renovation of your flat, the housing corporation has started cooperating with various organizations such as DOCK, the Neighborhood Team and Work & Income of the municipality of Utrecht, to not only renovate the homes, but also help the residents in other ways and with other things, for example, if people have debts, or to find a job.

What do you think of that?

What have you noticed of that so far? For example, have you been offered help or have you been in contact with someone from DOCK or the Neighborhood Team or Work and Income?

In what way have you received help? How or what did they help you with?

How has this affected how you feel or your life and health?

What influence has this had on how you experience the renovation?

Neighbourhood

I would also like to ask you a few questions about the neighbourhood where you live.

What do you think of your neighbourhood?

How would you describe the quality of the neighbourhood? What things do you like and dislike about the neighbourhood?

Are there certain problems in the neighbourhood that bother you?

How do these problems affect the way you feel and your health?

Green spaces

What do you think of the greenery in the area? (too much, too little, nice, not nice?)

Do you ever go to a park or other green spaces nearby?

If so, when, with whom, why do you do that, what kind of activities do you mainly do there?

How do you feel about being in green space or nature? What do you think of that?

How do green spaces affect the way you feel, your health, well-being, mood?

How nice it is to live in a certain place does not only have to do with what the place looks like, for example how clean the streets are and how much greenery there is, but also with the people who live there.

Do you often have contact with other people in the neighbourhood or in your flat/building?

What do you do together?

Do you feel connected to your neighbours in the flat/building or in the neighbourhood?

Other interventions/changes

Quite a lot is happening and changing in the neighbourhood. Renovating old flats, such as renovating your flat, is one thing, but there is more going on. For example, there is a plan to improve the greenery and green areas in the neighbourhood and to connect them more so that all kinds of green 'detours' are created in the neighbourhood. You may have heard about it. Work is also being done on a better connection with the forest just outside the neighbourhood. And elsewhere in the neighbourhood there is a high flat that is going to be demolished; new homes will be built, and the public space will be redesigned, so the neighbourhood will look different.

I'm curious whether you have heard anything about these changes or these plans? Have you heard or do you know anything about this?

What do you think of these changes or plans?

What do you think about the neighbourhood being renovated in different ways?

How do you think that affects the health of the people who live here?

Interview guide for interviews with residents *after* renovation of their own apartment

Introduction and demographics

Can you introduce yourself? Can you tell me something about yourself?

- How long have you been living here?
- Who do you live here with?
- Do you have a paid job and what kind of job do you do?
- How old are you?

Health – meaning and definition

The research we are doing is about how various changes in the neighbourhood, such as the renovation of your flat, influence the health of the people living here. Before we actually talk about the renovation, I am curious:

What do you understand by health? When I say the word 'health' what comes to your mind? It can be anything, you can just say what comes to mind.

When would you define someone as 'healthy'?

When would you define someone as 'unhealthy'?

How is your own health? Can you tell me something about that?

What do you do for your health, to stay healthy?

Renovation

Now let's talk about the renovation, because that is of course what you are dealing with now.

General

What do you think about old flats in the neighbourhood being renovated? What is your opinion about that?

What do you think of the renovation of your own flat?

Quality of and feeling about the house before and after renovation

What did you think of your house as it was before the renovation? How would you describe the quality of your home before the renovation? What did you like or dislike about your home?

Were there any problems in your home that bothered you or affected your health and how you felt?

How is that now? What do you think of your renovated home? How would you describe the quality of your renovated home as it is today? What do you like or dislike about your home as it is now?

Do you think differently about your home now compared to before the renovation? For example, are you more proud of your home since it has been renovated? Or less happy with certain things?

What changes do you especially notice in your home now, compared to what it was like before the renovation?

Is your house easier to keep clean since it has been renovated? What causes that?

Renovation period, renovation stress and the influence of renovation on health

How did you experience the renovation?

How stressful was/is the renovation for you? What things stressed you out the most?

During which period of the renovation were you most stressed? (e.g. during renovation of the front facade, renovation of the front and back of your own home, moving to a guest house during renovation of the inside of the home) Can you explain why you were mostly stressed during that period?

How stressful is your living situation right now? What things stress you out the most?

How does the renovation affect your health and how you feel?

Was that different during the renovation than now that your apartment has been delivered/finished? And what do you think this will be like when the entire flat/building is finished?

Did you feel that you were well informed during the renovation about when things were going to happen? Why or why not, can you explain?

Social interventions

For the renovation of your flat, the housing corporation has started cooperating with various organizations such as DOCK, the Neighbourhood Team and Work & Income of the municipality of Utrecht, to not only renovate the homes, but also help the residents in other ways and with other things, for example, if people have debts, or to find work. This is called 'social renovation'.

What do you think of that?

What have you noticed of this (social renovation)? For example, have you been offered help or have you been in contact with someone from DOCK or the Neighbourhood Team or the Work and Income department of the municipality? (Or perhaps you received extra help from the construction company or the housing corporation?)

In what way did you receive help? How or what did they help you with?

How did this affect how you felt/feel or your life and health?

What influence did this have on how you experienced the renovation?

Neighbourhood

I would also like to ask you a few questions about the neighbourhood where you live.

What do you think of your neighbourhood?

How would you describe the quality of the neighbourhood? What things do you like and dislike about the neighbourhood?

Are there certain problems in the neighbourhood that bother you?

How do these problems affect the way you feel and your health?

Green spaces

What do you think of the greenery in the area? (too much, too little, nice, not nice?)

Do you ever go to a park or other green spaces nearby?

If so, when, with whom, why do you do that, what kind of activities do you mainly do there?

How do you feel about being in green space or nature? What do you think of that?

How do green spaces affect the way you feel, your health, well-being, mood?

Social contact and social cohesion

How nice it is to live in a certain place does not only have to do with what the place looks like, for example how clean the streets are and how much greenery there is, but also with the people who live there.

Do you often have contact with other people in the neighbourhood or in your flat/building?

What do you do together?

Do you feel connected to your neighbours in the flat/building or in the neighbourhood?

Other interventions/changes

Quite a lot is happening and changing in the neighbourhood. Renovating old flats, such as renovating your flat, is one thing, but there is more going on. For example, there is a plan to improve the greenery and green areas in the neighbourhood and to connect them more so that all kinds of green 'detours' are created in the neighbourhood. You may have heard about it. Work is also being done on a better connection with the forest just outside the neighbourhood. And elsewhere in the neighbourhood there is a high flat that is going to be demolished; new homes will be built, and the public space will be redesigned, so the neighbourhood will look different.

I'm curious whether you have heard anything about these changes or these plans? Have you heard or do you know anything about this?

What do you think of these changes or plans?

What do you think about the neighbourhood being renovated in different ways?

How do you think that affects the health of the people who live here?

<u>Interview guide for professionals</u>

Introduction and demographics

First of all, could you briefly introduce yourself? What kind of work do you do / what is your position / at what type of organization?

Health

The study is about the effects of various interventions in the living environment in the neighbourhood on the health of residents. The social renovation of the [streetname] is one of these interventions. Before we really talk about social renovation, I would first like to briefly talk about health.

What do you understand by health? When I say the word “health,” what comes to your mind? It can be anything, you can just say what comes to mind.

When would you define someone as ‘healthy’?

When would you define someone as ‘unhealthy’?

How would you describe the health of residents in this neighbourhood?

Holistic housing renovation (‘social renovation’)

Now I would like to talk about holistic housing renovation.

What role do you/your organization play in holistic housing renovation?

What do you understand by holistic housing renovation? How would you define it?

What possible effects in general do you expect holistic housing renovation to have on residents?

What impact do you think holistic housing renovation has on the health of residents?

- And on the overall well-being of residents?

- In what way, via which parts of the intervention? Through what mechanisms?

Do you think that holistic housing renovation mainly has physical, mental or 'social' (well-being in general, social cohesion) health effects?

- What do you think are the effects of holistic housing renovation regarding the physical health of residents?

- What do you think are the effects regarding the mental health of residents?

- What do you think are the effects regarding the 'social' health (well-being in general, social cohesion) of residents?

How do you think the various components of holistic housing renovation influence the health of residents?

Are there parts that you think have more influence than other parts?

Which parts are those, and why specifically these?

Picture with mechanisms from holistic housing renovation to health (from literature review)

Various mechanisms can be identified from the literature on how a combination of physical renovation and social interventions can influence health. This picture shows several possible mechanisms. It shows how physical renovation can influence both physical and mental health, both positively and negatively, and how various social interventions can influence health (particularly mental health).

I am curious which mechanisms from this overview you recognize (especially) among residents, when you look at the holistic housing renovation on [street].

Can you give examples?

Are there also mechanisms that you do not recognize at all?

What are your expectations regarding these mechanisms for the residents of [street]?

Other interventions – expected health effects on residents

Holistic housing renovation is one thing in the lives of the residents, but there are more things going on in the neighbourhood (in addition to things in people's personal lives, of course). From the IGLO project, we are curious about how multiple, different interventions in the living environment of one neighbourhood jointly influence the health of residents. In addition to the renovation of the [street], we are investigating a number of other interventions in the neighbourhood, such as the demolition and new build of the 10-storey flat on the [street 2], and interventions in green spaces, which are intended to improve the greenery and green spaces and make them more attractive to residents of the neighbourhood, so that they will use them more. Consider the Green walking route, or improving

the accessibility of the forest nearby. For example, we also conduct research into the difference in need and use of greenery within the neighbourhood versus nature close by, such as the forest. I am curious whether these other changes in the neighbourhood also affect the residents of [street].

What do you notice about this among residents? Does it matter to them? Are they concerned with it? Or not at all? How do you notice that?

What are your expectations about how multiple interventions in the same neighbourhood can affect the health of residents?
