# Supplementary figures and images for: Health effects of holistic housing renovation in a disadvantaged neighbourhood in the Netherlands: a qualitative exploration among residents and professionals
Source: BMC Public Health. 2024 Apr 16;24:1056. doi: 10.1186/s12889-024-18500-2 (PMC11020185; doi:10.1186/s12889-024-18500-2)

Simplified visual display of the mechanisms from our realist review

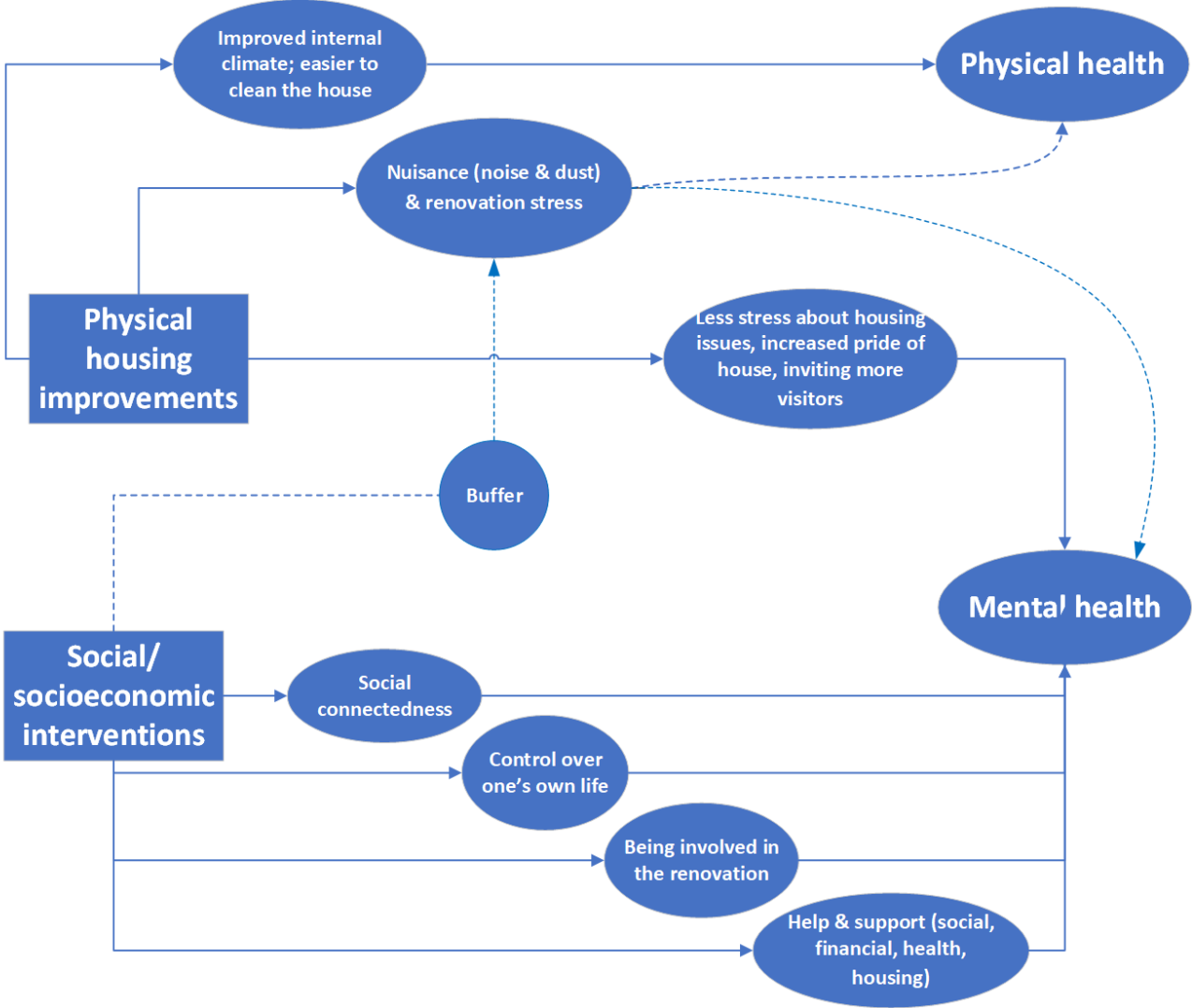

Supplement: Supplementary file 2 — Additional file 2. Simplified visual display of the mechanisms from our realist review. Simplified visual display of the mechanisms from our realist review Description of data: Simplified visual display of the mechanisms from our realist review, used in the interviews with professionals. [file 12889_2024_18500_MOESM2_ESM.pdf]
